# Automatic Compilation from High-Level Biologically-Oriented Programming Language to Genetic Regulatory Networks

**DOI:** 10.1371/journal.pone.0022490

**Published:** 2011-08-05

**Authors:** Jacob Beal, Ting Lu, Ron Weiss

**Affiliations:** 1 BBN Technologies, Cambridge, Massachusetts, United States of America; 2 Departments of Biological Engineering, Electrical Engineering and Computer Science, Massachusetts Institute of Technology, Cambridge, Massachusetts, United States of America; Tel Aviv University, Israel

## Abstract

**Background:**

The field of synthetic biology promises to revolutionize our ability to engineer biological systems, providing important benefits for a variety of applications. Recent advances in DNA synthesis and automated DNA assembly technologies suggest that it is now possible to construct synthetic systems of significant complexity. However, while a variety of novel genetic devices and small engineered gene networks have been successfully demonstrated, the regulatory complexity of synthetic systems that have been reported recently has somewhat plateaued due to a variety of factors, including the complexity of biology itself and the lag in our ability to design and optimize sophisticated biological circuitry.

**Methodology/Principal Findings:**

To address the gap between DNA synthesis and circuit design capabilities, we present a platform that enables synthetic biologists to express desired behavior using a convenient high-level biologically-oriented programming language, Proto. The high level specification is compiled, using a regulatory motif based mechanism, to a gene network, optimized, and then converted to a computational simulation for numerical verification. Through several example programs we illustrate the automated process of biological system design with our platform, and show that our compiler optimizations can yield significant reductions in the number of genes (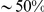) and latency of the optimized engineered gene networks.

**Conclusions/Significance:**

Our platform provides a convenient and accessible tool for the automated design of sophisticated synthetic biological systems, bridging an important gap between DNA synthesis and circuit design capabilities. Our platform is user-friendly and features biologically relevant compiler optimizations, providing an important foundation for the development of sophisticated biological systems.

## Introduction

Synthetic biology is an emerging field at the interface of biology, engineering, and physical sciences, which focuses on the systematic design and engineering of biological systems [1]–[3]. This field brings a set of new approaches for tackling biological problems and at the same time addresses real world problems. Building upon early studies such as an engineered genetic toggle switch [4] and an oscillator [5], synthetic biology efforts have created a large collection of functional devices and small regulatory modules that use a variety of biochemical processes. These efforts include both single cell and multicellular functions, such as logic function evaluators, an edge detector, synchronized oscillator, and spatial pattern generators [6]–[9]. Simultaneously, DNA synthesis technologies have demonstrated dramatic improvements over the past decade [10] with a recent publication of a functional synthetically synthesized mega-base-pair genome [11]. Based on these recent successes, synthetic biology promises to revolutionize biomedical, environmental, and energy-related areas.

While DNA synthesis has enjoyed remarkable progress recently and the number of publications of experimental systems is growing rapidly, the regulatory complexity of synthetic biological circuits published over the last few years has remained stagnant [12], [13]. It is still a daunting task to design and implement new regulatory networks that perform desired novel functions, and the creation of new genetic circuits is a tedious and time-consuming ad hoc manual process. Many challenges remain before we can achieve efficient and reliable construction of new functional circuits, including the availability of a large library of validated and well-characterized parts, readily available and efficient automation of DNA assembly of these parts, and better computational tools for predicting the behavior of circuits assembled from these parts within a host organism. As a result, engineering even the simplest circuits often requires years of hard work and constructing systems with multiple parts is very challenging.

To accelerate the realization of sophisticated synthetic biological systems, here we propose and analyze a new automated circuit design workflow that helps overcome the gap between idea and biological implementation. We provide a platform for biological system designers to express desired system functions using a user-friendly high-level biologically-focused programming language. Our compiler then transforms such a high-level design into a genetic regulatory network and optimizes it to conserve scarce biological resources (e.g. metabolic load, applicable BioBrick parts) [14], [15]. The resulting genetic regulatory network is simulated computationally and eventually realized in cells.

Our platform can be integrated with efforts that provide “assembly-language” level composition of synthetic biology elements and modeling, including biological modeling standards such as SBML [16], the Synthetic Biology Open Language (sbolstandard.org), and CellML [17], and means to simplify biological model building such as Antimony [18], little B [19], and ProMoT [20]. Other related circuit design tools, such as Clotho's Eugene [21] and GenoCAD [22] can be used to improve the process of analyzing and converting gene regulatory networks into physical manifestations through integration with automated DNA assembly protocols [23], [24].

**Figure 1:**
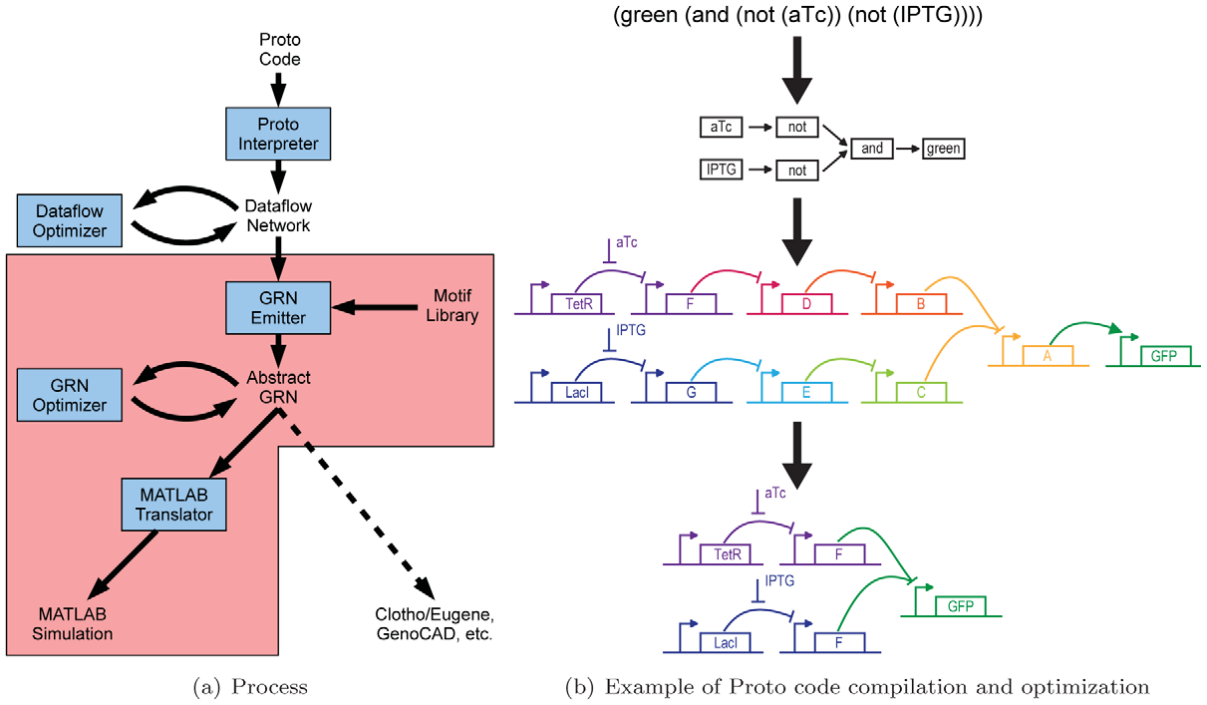
Proto biocompiler architecture and example. (a) This paper extends the Proto spatial computing language with mechanisms for genetic regulatory network design (pink). (b) An example showing how a simple high level behavioral specification is converted first into a dataflow network, then into a genetic regulatory network, and finally optimized. In this example, green fluorescence is turned ON only when both small molecule inputs aTc and IPTG are not present (aTc, anhydrotetracycline. IPTG, Isopropyl 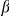-D-1-thiogalactopyranoside). A–F represent transcriptional repressors to be chosen later from a parts library.

We rely on our spatial computing language Proto [25] to serve as a plausible language for designing and implementing synthetic biological systems [26]. In previous work, we showed how to convert a high level description into a lower level genetic representation using manual transformations. We now describe a new automatic compilation technique for mapping a high-level behavioral description expressed in the Proto language into an abstract genetic regulatory network (a network with some elements left unspecified). Our compiler optimizes this gene network and generates a computer simulation of the optimized network (Figure 1). To achieve this new process for automated synthetic circuit design, we augmented the Proto language to support motif-based compilation and standardized device families. Our synthetic gene networks are organized into a series of promoter-genes-terminator *functional units*, where each such unit has a known input/output relation. Parts in these functional units can then be selected from a database of characterized DNA parts, such that they are compatible with one another with respect to their input/output thresholds and characteristics.

In the remainder of this manuscript, we begin with a description of the Proto programming language and its adaptation to biological system design. Then, we discuss genetic parts and device input/output requirements, followed by the description of our new tools for compilation and optimization of engineered systems. We then demonstrate and analyze the capabilities of this platform using a few examples that illustrate the power of the automated tools. Finally, we conclude by discussing the current abilities, limitations, and future directions of our synthetic biological design approach.

## Methods

### Proto programming language

**Figure 2:**
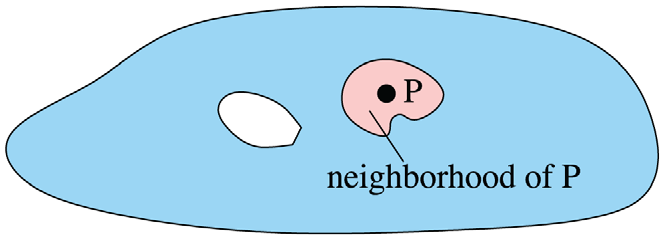
An amorphous medium is a manifold where every point is a universal computational device that knows its neighbors' recent past state.

One way to view biological systems is as a collection of computational elements (i.e. cells) distributed spatially. A promising approach to the challenges of distributed control over such elements is to focus on the continuous space that they occupy using the *amorphous medium* abstraction [25], [27]. An amorphous medium is a manifold with a computational device at every point in space, where every device knows the recent state of all other devices in its neighborhood (Figure 2). While an amorphous medium cannot, of course, be constructed, it can be approximated using a discrete network of spatially distributed computing devices. Our language, Proto, uses the amorphous medium abstraction to factor the distributed programming task into three loosely coupled subproblems: global descriptions of programs, compilation from global to local execution on an amorphous medium, and discrete approximation of an amorphous medium by a real network that consists of many elements.

Proto is a functional language that is interpreted to produce a dataflow graph of operations on fields; for the purpose of this paper, we assume that all function calls are inlined in the graph, though that need not be the case in general. This program is then evaluated against a manifold to produce a field of values that evolve over time. We call an elementary Proto operation a *primitive*. Proto uses four families of primitives: point-wise operations such as ‘+’ that involve neither space nor time, restriction operations that limit execution to a subspace, feedback operations that establish state and evolve it in continuous time, and neighborhood operations that compute over neighbor state and space-time measures, then summarize the computed values in the neighborhood with a set operation like integral or minimum.

With appropriate operators, compilation and discrete approximation are straightforward. Thus, Proto makes it easy for a programmer to carry out complicated spatial computations using simple geometric programs that are robust to changes in the network and self-scale to networks with different shape, diameter, density of nodes, and execution and communication properties [28], [29].

In [26], we demonstrated that we can encode a spatial program to obtain a bullseye pattern similar to our experimental work from [9] and then convert it manually to a genetic regulatory network. In this paper we consider several examples that do not have a spatial component yet but are handled entirely by our compiler.

### Genetic parts and input/output requirements

**Figure 3:**
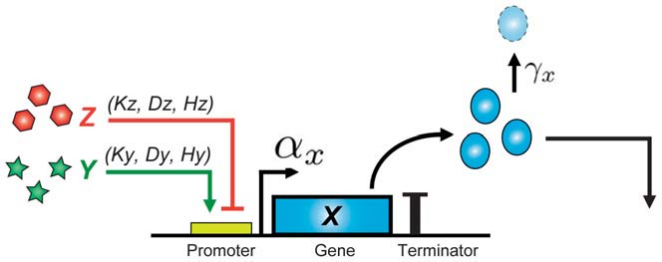
Our genetic regulatory network designs are based on promoter-gene-terminator functional units such as the example shown above. The regulatory gene 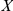 is regulated by upstream transcriptional activator 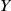 and transcriptional repressor 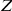, and produces proteins for downstream regulation. The parameters are defined in Section.

Biological organisms are sophisticated systems capable of efficient information processing and robust function with an underlying machinery that is based on regulatory networks comprising genes, proteins, and small molecules. Natural regulatory networks are often very complicated, such that for even the simplest functions many components are involved and entangled with each other. Engineered systems, on the other hand, are usually designed with simplicity in mind for ease of human comprehension and manipulation, sometimes at the expense of optimality. As is common in synthetic biology, we use a modular approach to the construction of new gene networks by assembling small functional parts and modules into a network of interconnected regulatory elements. In our design framework, each regulatory element is a *functional unit* consisting of a promoter, one or more genes, and a terminator (Figure 3). The gene is regulated, positively or negatively, by upstream elements whose concentration serves as the input signal. The promoter produces proteins as output that can serve as transcriptional regulatory factors inputs for downstream regulatory elements.

We describe the behavior of each regulatory element in the network using a potentially multi-input sigmoidal transfer curve. Our automatic compilation from high-level Proto code to gene networks composes together regulatory elements, thus creating an overall system that is a composition of these sigmoidal transfer curves.

**Figure 4:**
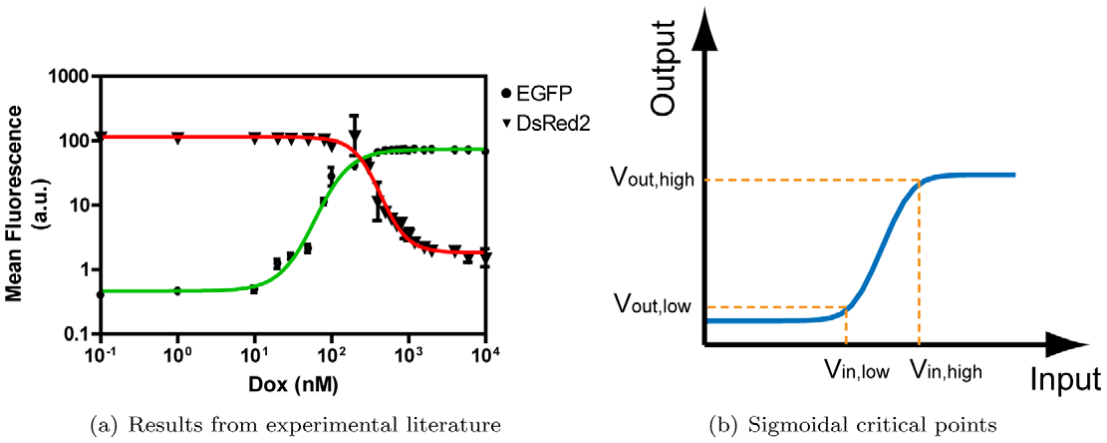
Transfer function experiments and requirements. Model and parameters are based on sigmoidal behaviors documented in the experimental literature, as in the graph from [30] shown in (a), showing sigmoidal responses of green and red fluorescence proteins upon Doxycycline (Dox) induction. In this network implemented in AINV15 cells, Dox binds rtTA and activates expression from the TRE promoter of an Enhanced Green Fluorescence Protein and mammalian-optimized LacI repressor. In turn, LacI represses production of DsRed2 from a Hef1a promoter engineered with lac operators. (b) Every sigmoidal curve has an input concentration window that results in large output variations. The curve shown is for transcriptional activation. Repression is represented by an analogous inverse sigmoidal curve.

To understand the feasible range of sigmoidal transfer curves, we can begin by analyzing experimental data for transcription factors (e.g. the repressor and activator shown in Figure 4(a), adapted from [30]). Alternate transfer curves can be obtained experimentally using a variety of genetic mutations [31], for example by incorporating various protein decay tags [32], adjusting ribosome binding efficiencies [33] and integrating promoters with different strengths [34]. For every sigmoidal input-output curve we define a transition window for the input level input 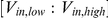 where modulation of input results in significant changes in output in the range 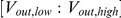 (illustrated for the case of an activator in Figure 4(b)). Outside of this window, however, output is relatively insensitive to input fluctuations, owing to the sigmoidal shape of the transfer curve.

This behavior allows us to categorize the different regimes of an input signal based on how they induce different output responses, in a manner that is analogous to standardized design of electronic digital components. In this design methodology, insensitivity to fluctuating input signals at the low and high ends is employed to maintain the digital abstraction reliably, i.e. circuit output values exhibit either low or high levels as appropriate, but not intermediate levels (after allowing for signal propagation and transition delays). The output of each functional unit depends on its upstream inputs and often simultaneously serves as an input for regulation of downstream units.

We thus require functional units that all implement *signal restoration*, where 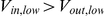, 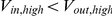 and input levels below 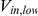 or above 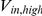 always result in output levels below 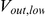 or above 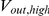. As such, the output of any regulation unit in this family is always a better representation of a digital value than the input, and when connected together these units implement digital computing. For an introduction to digital logic design and why this methodology enables construction of large scale reliable systems, see e.g. [35]. Note that we ultimately seek to create hybrid digital/analog circuits, for example ones that integrate gene regulatory modules with digital [36] and analog [37] behaviors, and that function reliably in noisy biological environments. In other efforts (e.g. [38]), we have presented methods for obtaining desired analog functions in synthetic gene networks using control theory methodologies of safety and reachability analysis. Likewise, electronic circuit abstractions for modeling and control of circuit dynamics might be adapted to allow automated design of biological circuits with complex dynamic behavior, such as oscillators or sequential developmental processes. But here we focus on automated digital logic synthesis for steady-state behavior.

One way of describing the kinetics of such a regulatory element is with an ordinary differential equation. For purposes of this paper, we consider only natural or engineered regulatory elements with an appropriate sigmoidal behavior: high slope and large difference between high and low expression levels. For these elements, the ODE approximation is adequate, although in the future we plan to consider stochastic models as well. Stochastic models may allow elements with lower slope or less difference between high and low expression levels to be used, because they will allow more precise predictions of system behavior.

**Figure 5:**
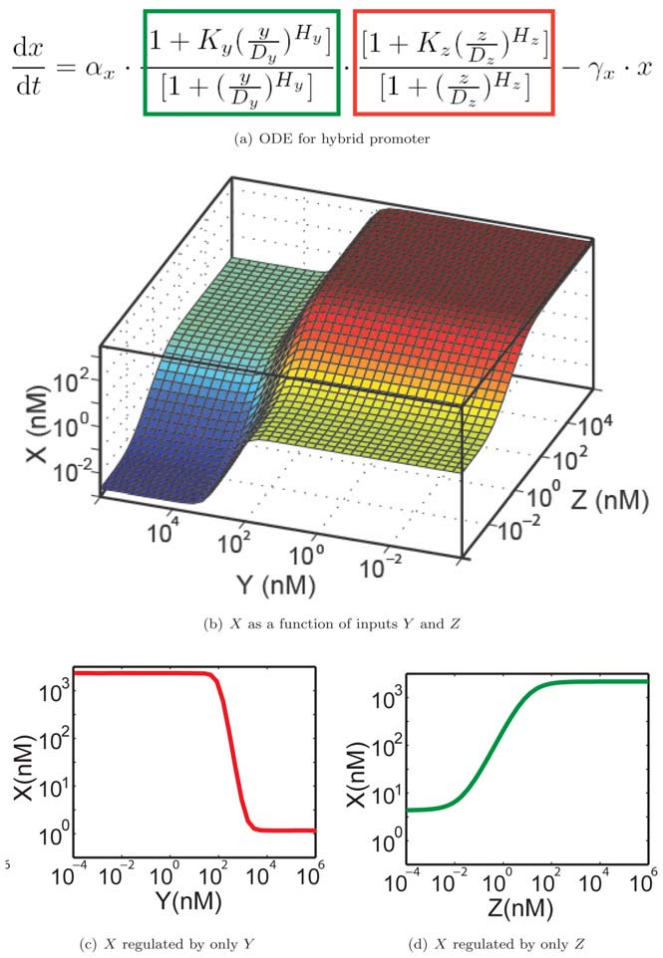
Transfer function models. Mathematical representation of the functional unit in Figure 3, via (a) ordinary differential equation that describes the kinetics of the transcription factor 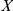. (b) The 3D profile of protein 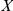 as a function of inputs 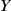 and 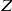. (c,d) Typical input-output relations of the functional unit when modulating only one of the inputs.

Figure 5 illustrates the ordinary differential equations used to model a hybrid promoter. Here, 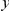 and 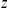 are concentrations of transcriptional activator 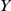 and transcriptional repressor 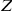 that regulate expression of protein 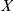 whose concentration is denoted by 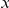. Basal expression of 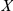 is 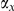 in the absence of 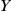 and 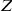 but is shifted in a sigmoidal fashion in the presence of either or both input signals. This is indicated by the second and third portions of the first term in the right hand side of the equation (green and red boxes) [39]. 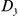 is the dissociation constant of transcription factor 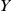 from the promoter, 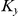 is the multiplicative activation change upon full induction by 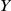, and 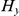 is the Hill coefficient representing the cooperativity of activation. 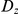, 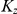, and 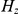 represent the corresponding effects of transcriptional repressor 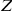. In addition, degradation term 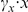 reflects the overall effect from direct degradation and decay of 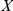 and dilution due to cellular growth.

The combination of degradation and sigmoidal production relations often give rise to a steady state input-output profile with multiple plateaus. Figure 5(a) illustrates a 3D concentration profile of 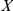 as a function of the levels of transcription factors 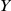 and 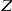. In this particular case, 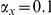 nM/min, 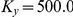, 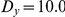 nM; 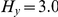, 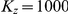, 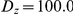 nM, 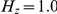, and 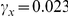 nM/min. Figures 5(b) and 5(c) show slices along the direction perpendicular to the 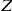 axis and 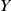 axis, respectively. These parameters are taken from a range of plausible values established by prior experimental results [9], [40]. A network that consists of such regulation units can be characterized by an array of parameter sets 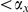,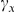,<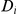,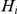,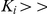, where the transfer curves influence one another according to the interconnection between the regulation units.

**Figure 6:**
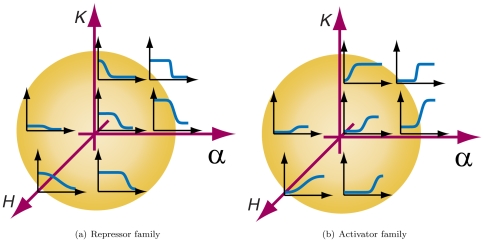
Diagrammatic representation of representative variants for repressors and activators along parameters 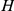, 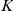, and 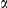 characterizing the behavior of the sigmoidal curve. Curves in different insets correspond to their specific position in this space.

A challenge to implementing such designs in a biological setting is to obtain or engineer a large enough set of regulatory parts that exhibit compatible transfer curves. Figure 6 shows diagrammatic parameter spaces for 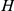, 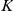, and 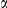 that demonstrate representative behaviors of different variants, and how experimental adjustment of parameters may be able to modify the transfer curve of an existing combination of regulatory elements to create functional units that have compatible signal thresholds. In the remainder of this manuscript we use only one parameter set for all functional units. However, all digital designs produced by our automatic compilation approach will operate correctly when instantiated using any set of orthogonal regulatory units that all satisfy the signal restoration requirements above.

To create a sizable library of such compatible regulatory elements, we would mutate various genetic aspects of existing regulation devices to achieve desired transfer curves. For example, modifying transcription and translation rates allows us to affect 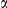, modifying protein half lives affects 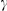, and altering transcription factor DNA binding affinity alters 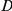. As we and others have previously demonstrated, these can all be modified experimentally [9], [31], [37], [41].

Concentration thresholds that ensure signal restoration can be established for other types of regulatory interactions as well. For example, it is often useful to incorporate detectors for various small-molecule signals into engineered circuits, such as using the transcriptional repressor TetR to detect high concentrations of aTc. A mathematical model for such a regulation unit, comprising TetR and its corresponding inducer aTc, with explicit description of the two different conformational states of the repressor, is defined as follows:
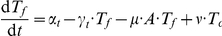


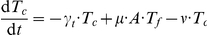


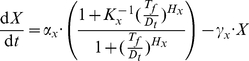
(1)where 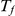 is the free form of repressor TetR, 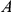 refers to inducer aTc, 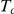 is the TetR-aTc complex which does not bind DNA, 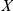 is a protein whose expression is regulated by TetR promoter, and 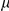 and 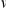 are the rate constants for TetR / aTc association and dissociation reactions (i.e. 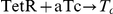 and 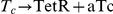). Because aTc has a sigmoidal interaction with TetR, we can set 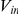 and 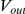 levels for aTc just as we would for a regulatory protein.

### Motif-based compilation and optimization

Using device standards such as the ones defined in the previous section, we can transform Proto programs into genetic regulatory networks by a process of motif-based compilation. The resulting designs are then optimized using various forms of standard computer code optimization techniques that we adapted for the biological milieu.

**Figure 7:**
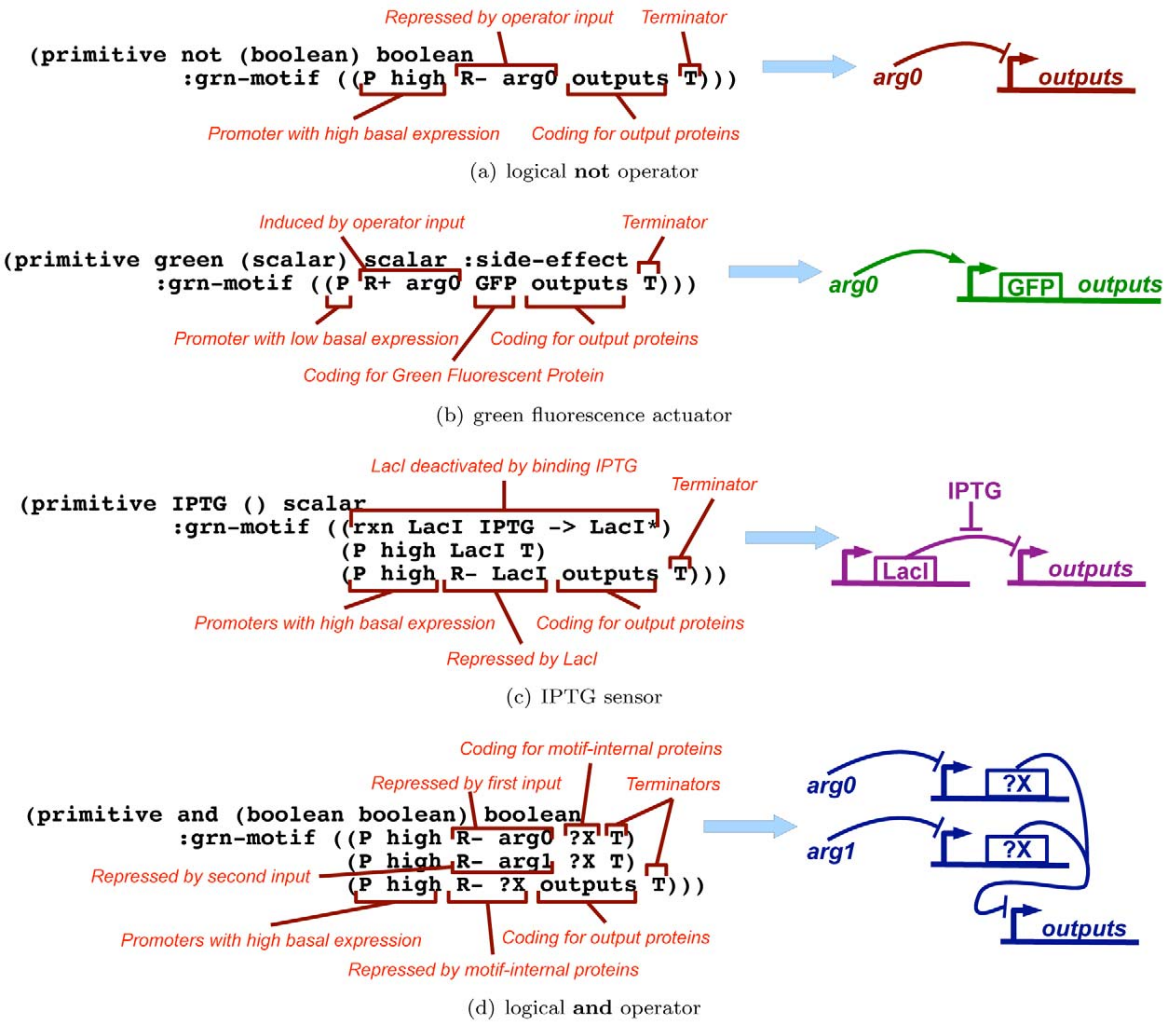
Example of BioCompiler motif declarations. (a) Logical **not** operator, (b) Green fluorescence actuator, (c) IPTG sensor and (d) A non-branching logical and operator. Terminators are not shown in the gene network diagrams for simplicity.

The compilation process relies on associations between each Proto primitive and a genetic regulatory network fragment. These associations are declared in Proto as annotations on primitives. For example, the logical **not** operator is associated with a biological inverter motif by the statement shown in Figure 7(a). The first line declares the **not** operator as a primitive with a boolean input and a boolean output. The second line contains a corresponding description of a functional unit for a genetic regulatory network, in this case a strong constitutive promoter repressed by a protein given the local identifier arg0, which represents the **not** operator's input. This is followed by coding regions for the protein outputs (each of which is implicitly fused to a ribosome binding site), then finally a transcriptional terminator.

Motifs can include many other element types. For example, a motif can specify particular molecules to be used, as in the case of the **green** actuator shown in Figure 7(b), where a green fluorescence ‘side-effect’ is implemented by the inclusion of a Green Fluorescent Protein (GFP) coding region in the motif. Motifs can also include reactions with small molecules, as in the case of an **IPTG** sensor shown in Figure 7(c), which is based on LacI derepression to detect the presence of the small-molecule signal IPTG. Motifs can include internal signalling variables that are filled in by the compiler when needed, as in the case of the **and** operator shown in Figure 7(d) where an appropriate transcriptional repressor will be automatically selected. This motif implements a non-branching logical and using inverted input to a nor gate.

In order to transform a Proto dataflow computation into an abstract genetic regulatory network, the compiler first maps each operator to its associated motif and each dataflow edge and internal motif variable to a regulatory protein. These motifs and proteins are then linked together, using the structure of the dataflow graph, to form an abstract genetic regulatory network. The particular choice of molecules and sequences to implement this network is not fully determined at this point, but left for a later stage of compilation, such as might be provided by a system like GEC [24], Eugene [21], or Matchmaker [42], with part selection guided by the standards from Section 0. An initial set of target rate constants for the network (to be modified as the implementation is determined) are filled in from the motifs where specified and left as symbolic values to constrain part selection where not specified.

**Figure 8:**
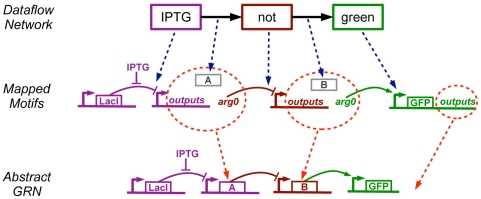
A Proto dataflow computation is compiled to an abstract genetic regulatory network in two stages. First, each operator is mapped to a motif and each dataflow edge is mapped to a regulatory protein (blue dotted lines). These elements are then linked together using the structure of the dataflow graph to form an abstract genetic regulatory network (red dotted lines).

For example, Figure 8 shows the transformation of a program for cells that fluoresce green when IPTG is not present, expressed in Proto as **(green (not (IPTG)))**. This program is interpreted to produce a dataflow computation on three operators, which are then mapped to the motifs specified by the declarations in Figure 7. The dataflow edges are assigned to yet-to-be-determined regulatory proteins 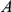 and 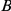 (there is no edge leading out of the **green** operator). The downstream motifs set the protein types, such that 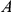 is a repressor and 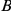 is an activator.

At this point we have obtained a genetic regulatory network that implements our high-level regulatory program, though it is still unoptimized and may be extremely inefficient. As we have demonstrated manually in [26], standard code optimization techniques, such as copy propagation, dead code elimination, and algebraic simplification, can be adapted to operate such on genetic regulatory networks.

For this paper, we automate the application of four simple but high-impact optimizations: copy-propagation, dead code elimination, double-negative elimination, and common subexpression elimination. Copy propagation tests whether a protein is used only to copy a value; if so, the original input may be used directly rather than the copy. Dead code elimination tests whether a regulatory protein is being used anywhere; if not, its production may be eliminated. Dead code elimination also disposes of functional units with no products. Double negative elimination searches for sequences of two inverters and excises them out of the network. Finally, common subexpression elimination searches for certain parallel constructions and collapses them into a single instance.

**Figure 9:**
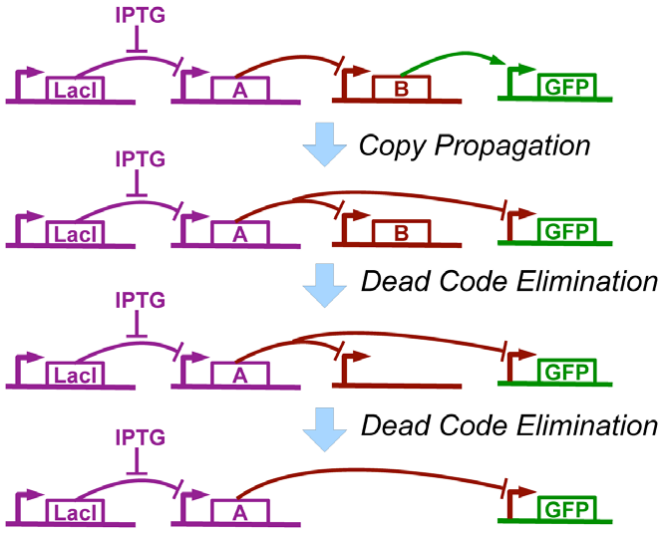
Example of optimization, applied to the compiled genetic regulatory network from **Figure 8**. Copy propagation changes GFP to be repressed by 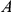 rather than activated by 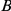, then dead code elimination removes first 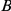 and then the regulatory region where 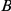 was formerly produced.

For example, in the case of our **(green (not (IPTG)))** program, copy propagation changes the input of the GFP-expression regulatory region from 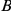 to 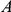. This then leaves 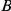 not regulating anything, so it is deleted, leaving a regulatory region that produces nothing, which is also deleted. Optimization thus reduces the number of unique promoters from 4 to 3, a 25% improvement (Figure 9).

In summary, by assigning genetic regulatory network motifs to Proto operators, we can automatically transform a Proto dataflow computation into an abstract genetic regulatory network, and the resulting genetic regulatory network can then be optimized using adapted forms of standard code optimization techniques. As we have shown in [26], the Proto language can be used to express more sophisticated programming constructs than the ones described in this section (e.g. multicellular spatial operations), and our compilation techniques can be applied to these as well.

## Results

To evaluate the behavior of our compiler, we tested it against a set of example programs and validated the behavior of each program in simulation. We analyzed the genetic regulatory networks generated by the compiler, both optimized and unoptimized, to determine improvements due to optimizations.

**Figure 10:**
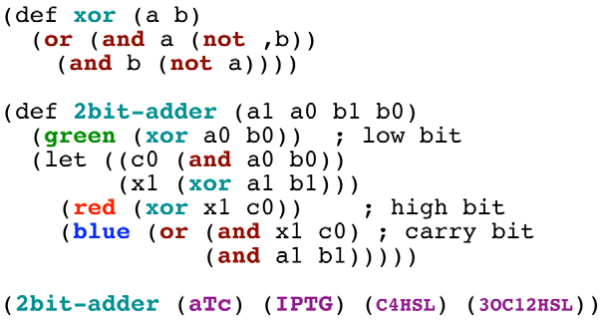
Proto code for a two-bit adder, showing operators in color. Inputs are purple, logic operators are red, functions are blue-green, and outputs are in their corresponding color.

We examine four example programs, three simple and one complex (logic truth tables shown in Tables 1, 2, 3 and 4). The example programs are:

- The single-NOT example used above: (green (not (aTc))).
- The three-gate example from Figure 1: (green (and (not (IPTG)) (not (aTc)))).
- A cascade of four NOT operations: (green (not (not (not (not (aTc)))))).
- A two-bit digital adder, which is a digital computation that takes as input two 2-bit numbers and outputs three bits–the sum of the inputs modulo four, and a carry bit if the sum is four or greater. The code for this program is shown in Figure 10.

**Table 1:** Input/output logic table for single-not system.

aTc	GFP
low	HIGH
HIGH	low

Entries on the left are inputs, and entries on the right are outputs.

**Table 2:** Input/output logic table for three gate system.

aTc	IPTG	GFP
low	low	HIGH
low	HIGH	low
HIGH	low	low
HIGH	HIGH	low

**Table 3:** Input/output logic table for quad-not system.

aTc	GFP
low	low
HIGH	HIGH

**Table 4:** Input/output logic table for 2-bit adder system.

aTc	IPTG	C4HSL	3OC12HSL	CFP	RFP	GFP
*A1*	*A0*	*B1*	*B0*	*Carry*	*X1*	*X0*
low	low	low	low	low	low	low
low	low	low	HIGH	low	low	HIGH
low	low	HIGH	low	low	HIGH	low
low	low	HIGH	HIGH	low	HIGH	HIGH
low	HIGH	low	low	low	low	HIGH
low	HIGH	low	HIGH	low	HIGH	low
low	HIGH	HIGH	low	low	HIGH	HIGH
low	HIGH	HIGH	HIGH	HIGH	low	low
HIGH	low	low	low	low	HIGH	low
HIGH	low	low	HIGH	low	HIGH	HIGH
HIGH	low	HIGH	low	HIGH	low	low
HIGH	low	HIGH	HIGH	HIGH	low	HIGH
HIGH	HIGH	low	low	low	HIGH	HIGH
HIGH	HIGH	low	HIGH	HIGH	low	low
HIGH	HIGH	HIGH	low	HIGH	low	HIGH
HIGH	HIGH	HIGH	HIGH	HIGH	HIGH	low

Italics show the role of each molecule, per Figure 10.

**Figure 11:**
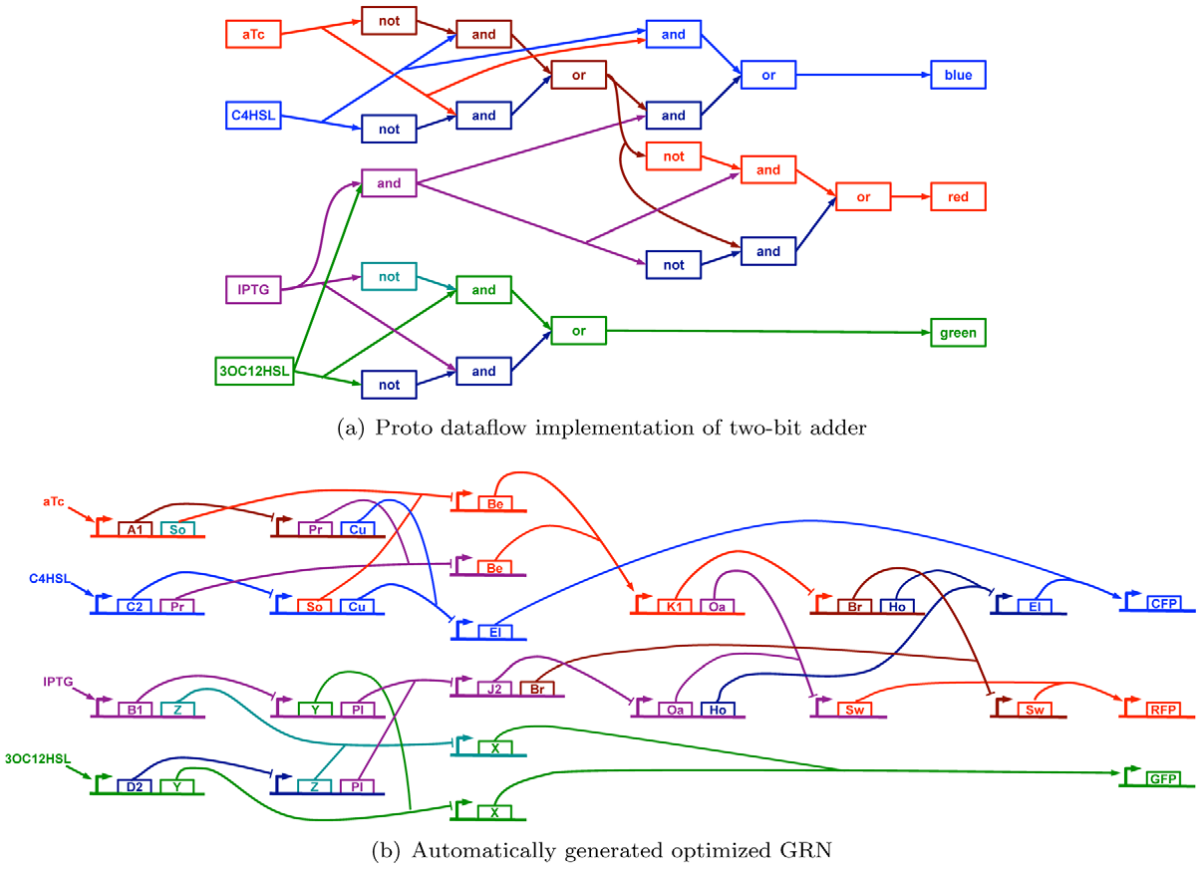
Large-scale example of Proto motif-based compilation: (a) a two-bit adder program, interpreted into a Proto computation and (b) transformed into an optimized genetic regulatory network (GRN) which is approximately half the size of the original network. The image is color coded to distinguish crossing edges; small-molecule binding reactions are elided. Note that although in this case the initial gene network has a one-to-one mapping between Proto operations and regulatory proteins, the final implementation logic is largely but not entirely inverted.

The two-bit adder, while not necessarily practical in a biological setting, is an example of a moderate scale program complex enough such that designing an optimized genetic regulatory network that implements this function presents a considerable challenge to a human. Figure 11 shows the Proto dataflow computation that the compiler produces for the 2-bit adder program, as well as the final optimized genetic regulatory network that the compiler generates. The resulting network is of significant scale and entangled complexity. But more interestingly, although a one-to-one mapping between Proto operations and regulatory proteins is used to generate the initial gene network, the internal logic of the optimized gene network is often inverted relative to the original dataflow network.

Given an optimized genetic regulatory network, the next task is to generate a simulation that can be used for validation. For this purpose, for each of the internal regulatory units in the network we use a single set of parameters that produce digital behavior with the same input and output range. Parameters for these parts are based on our earlier experimental results with synthetic gene networks [9], [40] with minor adjustments that are realistic given our experimental experience and that of others with modulating input/output characteristics of synthetic biological devices (e.g. [9], [31], [37], [41], [43]). We adapt the parameters from Section 0 to produce a device that can satisfy the I/O requirements such that the Hill coefficient and 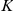 for activators is set to be the same as for repressors, 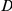 for activators is changed from 10 nM to 100 nM and 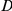 for repressors is changed from 100 nM to 25 nM, and 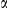 is changed from 0.1 nM/minutes to be 0.04 nM/minute. Each of these new values is well within the envelope of feasibility, and most represent rather minor changes.

**Figure 12:**
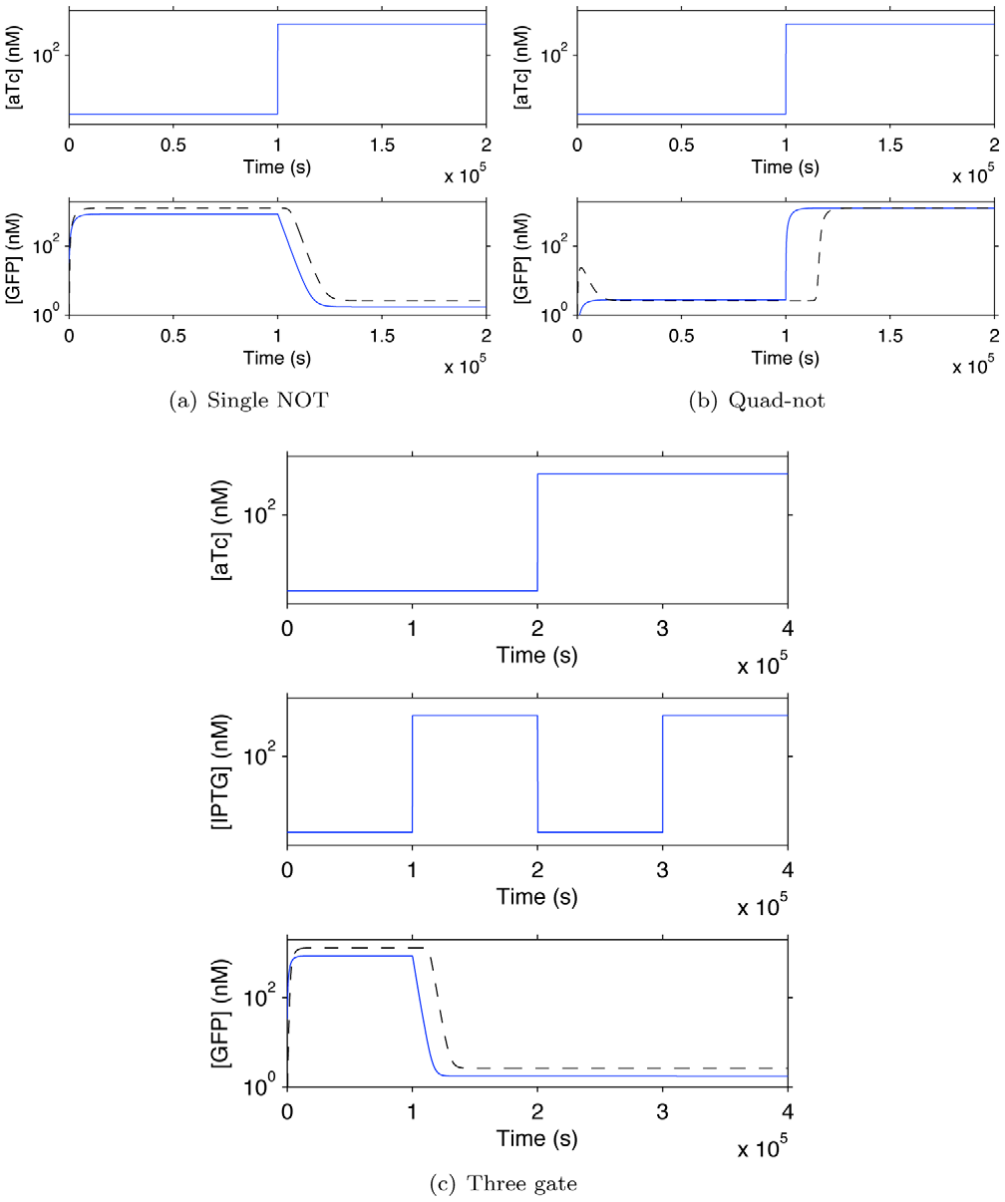
Simulation of automatically generated genetic regulatory networks executing for single-not, three-gate, and quad-not programs. The upper graphs for each network show small-molecule input concentrations and the bottom graphs show output GFP concentrations for the optimized (solid blue) and unoptimized (dashed black) networks.

**Figure 13:**
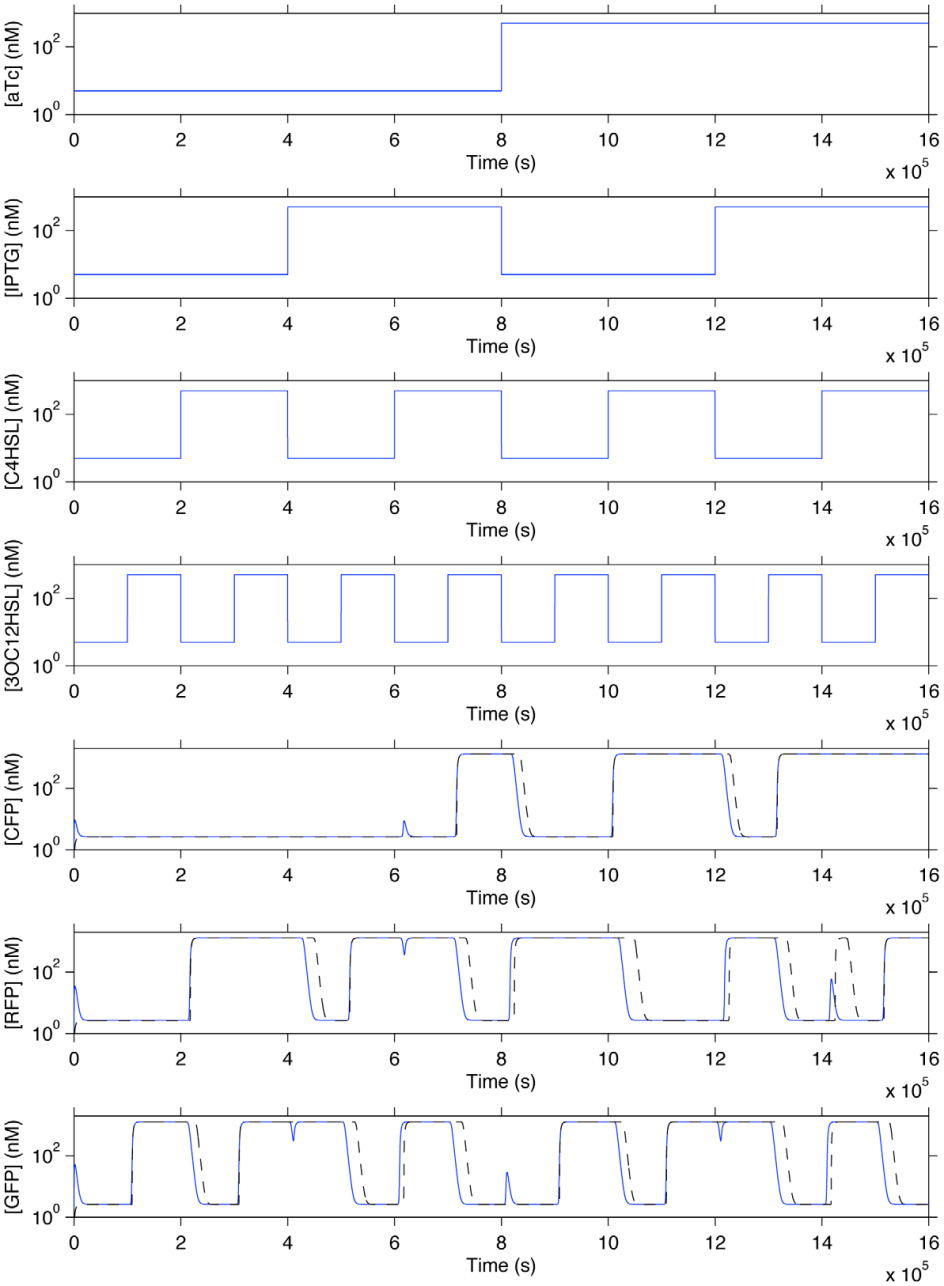
Simulation of automatically generated genetic regulatory networks for the two-bit adder. The upper graphs show small-molecule input concentrations and the lower three graphs show output CFP, RFP, and GFP concentrations for the optimized (solid blue) and unoptimized (dashed black) networks.

Once a gene network has been produced, the compiler emits a set of Matlab files containing a biochemical simulation of the specified network, using the reaction models presented in Section. Our simulations are based on ODE models with integration using a standard ode15 s Matlab stiff ODE-solver. For each system, we validate behavior by controlling each small-molecule input signal in the system. Our current simulations skip the small molecule induction step and directly simulate the activity of the small molecule regulated protein, but the next version of the compiler will also generate the small molecule interactions explicitly. The simulations test every combination of binary high and low levels for the inputs of the network, with each test lasting 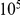 simulated seconds. The simulations are expected to produce the logical behaviors specified in Tables 1, 2, 3 and 4. Figure 12 and Figure 13 show the behavior of each network, demonstrating that the simulated genetic regulatory networks correctly implement the specified high-level programs. Note that, as expected during the operation of logic networks, transient glitches and hazards appear during transition periods until the system settles into steady state. The worst glitch takes place with the RFP output in the unoptimized 2-bit adder shortly after 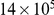 seconds. But importantly, the steady state behavior of optimized and unoptimized networks is equivalent.

We also analyzed the efficacy of optimization by comparing the structure of the optimized and unoptimized networks for each of our four test systems. Results are summarized in Table 5, showing significant improvements in every system. The results with the 2-bit adder are especially encouraging, demonstrating that our adaptation of classical optimization techniques are likely to be applicable with high efficacy across a broad range of possibly very complex programs.

**Table 5:** Optimization results for the four test systems.

		Proteins	Functional	Promoters	Delay
			Units	(Repressed/Activated/Constitutive)	Stages
Single-Not	Unoptimized	4	4	4 (2/1/1)	3
	Optimized	3	3	3 (2/0/1)	2
	% Improvement	25%	25%	33%	33%
Three-Gate	Unoptimized	10	10	9 (7/1/1)	5
	Optimized	4	5	4 (3/0/1)	2
	% Improvement	60%	50%	55%	60%
Quad-Not	Unoptimized	7	7	7 (5/1/1)	6
	Optimized	2	2	2 (1/0/1)	1
	% Improvement	71%	71%	71%	83%
2-bit adder	Unoptimized	55	56	53 (37/15/1)	12
	Optimized	26	23	24 (19/4/1)	7
	% Improvement	52%	59%	55%	42%

## Discussion

In this paper, we present a platform that allows synthetic biologists to design biological systems using the Proto high-level biologically-oriented programming language. To achieve the promise and potential of synthetic biology, the impressive recent advances in DNA synthesis and assembly capabilities must be matched with analogous advances in our ability to design sophisticated and reliable biological systems. Our platform is an important step towards this goal, providing synthetic biologists with a convenient mechanism to express sophisticated behavior, and a compiler that automatically transforms these programs into gene regulatory networks, optimizes these gene networks, and then initiates simulations of these optimized networks to validate their correct behavior. As shown in the examples in Section, our compiler is able to achieve significant reductions in the complexity of several engineered gene networks while preserving and even improving their function.

While our first automated version of the Proto platform already provides important functionality, there are still many challenges and further developments are needed. For example, the compiler should ensure that an engineered system is biologically feasible, i.e. the chosen regulatory parts comprising the complex system have parameter values within ranges achievable in living organisms and that these parts are compatible in terms of input-output levels. Experimentally this can be achieved by manipulating appropriate physical parameters, such as adding degradation tags for tuning protein decay rates and altering ribosome binding sites to modulate protein expression rates [32], [33]. Ultimately, the compiler would be able to obtain information about parts by accessing curated libraries, such as the Registry of Standard Biological Parts [15]. With this initial collection in place, it may be desirable to improve certain properties of particular parts, for example, increasing Hill coefficients to obtain better ultrasensitive all-or-none responses. It would also be valuable to have a library of useful and commonly found naturally occurring motifs, such as feed-forward loops, oscillators, bistable switches, and ultrasensitive cascades [44]. Note that our compiler is not restricted to digital logic, and future versions will incorporate analog, temporal, and feedback control. We also plan to incorporate other forms of compiler optimizations to further increase the efficiency of the automatically generated gene networks. These will be based both on standard computer code optimization techniques (e.g. constant elimination and algebraic simplification) as well as techniques that are specific to the biological substrate (e.g. incorporation of hybrid promoters and chimeric proteins).

Our current simulations are based on deterministic evaluation of the engineered biological networks. However, real genetic networks are intrinsically stochastic with fluctuations arising from biochemical events underlying gene expression and other biochemical processes [45]–[49]. Hence it is necessary that we obtain more precise characterization of noise margins and signal restoration in order to guarantee the correct operation of our engineered systems. For this purpose, the next version of our platform will also incorporate stochastic simulations [50], [51]. Stochastic behavior is usually regarded as a phenomenon that perturbs a system out of a desired operational range and hence possibly out of the proper functional regimes. To address such concerns may require the compilation of mechanisms that enhance the robustness of a system and its ability to attenuate noise. Other confounding elements include the time delays and structural considerations inherent in transcriptional regulatory systems [52], [53], as well as complex interactions with the cellular context, such as feedback in inducer uptake [53].

An important question to explore is the tradeoff between system simplicity and the incorporation of complex mechanisms to enhance robustness, and how such tradeoff can be presented to the system designer. Alternatively, one can also envision situations where noise inherent to biological systems could actually be exploited to increase the robustness of the system by adding heterogeneity where it is beneficial. It would be interesting to explore high level programming abstractions that can support such design principles. In all cases, however, we expect that the automatic compilation techniques presented in this paper will provide a useful base for future advances.
